# Fluorescent Sensor Arrays Can Predict and Quantify the Composition of Multicomponent Bacterial Samples

**DOI:** 10.3389/fchem.2019.00916

**Published:** 2020-01-15

**Authors:** Denis Svechkarev, Marat R. Sadykov, Lucas J. Houser, Kenneth W. Bayles, Aaron M. Mohs

**Affiliations:** ^1^Department of Pharmaceutical Sciences, University of Nebraska Medical Center, Omaha, NE, United States; ^2^Department of Pathology and Microbiology, University of Nebraska Medical Center, Omaha, NE, United States; ^3^Fred and Pamela Buffett Cancer Center, University of Nebraska Medical Center, Omaha, NE, United States; ^4^Department of Biochemistry and Molecular Biology, University of Nebraska Medical Center, Omaha, NE, United States

**Keywords:** multiparametric sensing, 3-hydroxyflavone, ESIPT, pathogenic bacteria, discriminant analysis, machine learning, pattern analysis

## Abstract

Fast and reliable identification of infectious disease agents is among the most important challenges for the healthcare system. The discrimination of individual components of mixed infections represents a particularly difficult task. In the current study we further expand the functionality of a ratiometric sensor array technology based on small-molecule environmentally-sensitive organic dyes, which can be successfully applied for the analysis of mixed bacterial samples. Using pattern recognition methods and data from pure bacterial species, we demonstrate that this approach can be used to quantify the composition of mixtures, as well as to predict their components with the accuracy of ~80% without the need to acquire additional reference data. The described approach significantly expands the functionality of sensor arrays and provides important insights into data processing for the analysis of other complex samples.

## Introduction

Reliable and rapid identification of pathogenic microorganisms in clinical laboratories is of high importance for the safety and health of the society (Doggett et al., 2016). Currently used methods are mostly based on PCR and mass-spectroscopy techniques, are time-consuming, and equipment-demanding (Váradi et al., 2017). Some rapid detection approaches reported in recent years use antibodies or aptamers to provide selectivity and specificity (Kubicek-Sutherland et al., 2017; Leonard et al., 2018). Sensor arrays are cross-reactive and not intrinsically selective, but they are often based on stable small molecules and provide more flexibility (Geng et al., 2019; Li et al., 2019). Several such systems were reported for successful analysis of bacteria (Phillips et al., 2008; Han et al., 2017). Although pathogen-associated biomarker tests such as ELLecSA (Cartwright et al., 2016) are promising, they are based on engineered enzymes, lack specificity, and are not able to identify particular species of pathogenic bacteria (Sheldon, 2016). Moreover, reliable analysis of mixed bacterial infections in clinical samples still represents a significant challenge (Laitinen et al., 2002; Kommedal et al., 2008).

In our previous study (Svechkarev et al., 2018b), we showed that the dataset containing responses from eight pure bacterial cultures can provide information beyond traditional species classification. In addition, the sensor was able to predict the Gram status of unknown samples outside of the training dataset. In the present communication, we expand the functionality of our sensor array by demonstrating its ability to analyze and quantify individual components of mixed bacterial samples. Importantly, the described approach can be generally applied to any data obtained using a sensor array and could aid in extraction of additional information about the analyte without the need of additional measurements.

## Materials and Methods

N-hydroxysuccinimide (NHS), 1-ethyl-3-(3-dimethylamino) propyl)carbodiimide (EDC), pyrenebutyric acid, 1,3-propyldiamine, 2-hydroxyacetophenone and 2-hydroxynaphthophenone were purchased from Sigma-Aldrich (St. Louis, MO). N,N-dimethylformamide (DMF), dimethyl sulfoxide (DMSO), sodium methylate, hydrogen peroxide (30%), N,N-dimethylaminobenzaldehyde, N,N-diphenylaminobenzaldehyde, and hydrochloric acid (Certified ACS Plus, 36–38%) were purchased from Fisher Scientific (Pittsburgh, PA). Ethanol was purchased from UNMC internal supply. Sodium hyaluronate (HA) was purchased from Lifecore Biomedical (Chaska, MN).

### Synthesis of the Dyes

The fluorescent ratiometric dyes used as reporters in the sensor array were synthesized following a modified Algar-Flynn-Oyamada procedure, as described elsewhere (Klymchenko et al., 2003). In brief, a corresponding para-substituted benzaldehyde is first reacted with an equimolar quantity of 2-hydroxyacetophenone in DMF in the presence of sodium methylate. The resulting chalcone is then oxidized with an excess of hydrogen peroxide in ethanol in presence of sodium methoxide. Detailed synthetic procedure for each dye are described in the Supplementary Material.

### Polymer Synthesis and Nanoparticle Loading

Amphiphilic hyaluronic acid (HA) polymers were synthesized as described in previous reports (Svechkarev et al., 2018a,b). Briefly, 40–45 mg HA (MW = 10–20 kDa) was dissolved in 1:1 ultrapure water and DMF along with 30 mg of NHS and 30 mg of EDC. After mixing for 30 min to activate the HA carboxylic acid groups, 10 weight percent of aminopropyl-pyrenebutanamide was added to the HA solution and allowed to react for 24 h. The reaction mixture was then removed and placed in 3,500 MWCO dialysis tubing and dialyzed against 1:1 water and ethanol for 4 exchanges over 24 h, then against pure water for 8 exchanges over 48 h to remove any impurities. Finally, the product was frozen and freeze-dried for later use. Stock solutions containing 60 mg of pyHA in 40 mL of ultrapure water, as well as 3 mg of each dye (in 10 mL DMSO) were prepared. Solutions of the polymer and dyes were mixed together (10 mL of the modified HA solution + 10 mL of the dye solution) to obtain 4 systems containing every dye mixed with the polymer. The final solutions were thoroughly mixed on a vortex mixer and loaded into the 3,500 MWCO dialysis bags. Samples were then dialyzed against ultrapure water with 8 exchanges over 48 h. After dialysis, the samples were purified using PD-10 columns, then frozen and freeze-dried for further storage at −20°C.

### Bacterial Culture, Staining, and Spectroscopy

Eight different bacterial species from our lab collection were used in this study, including four Gram-positive (*Staphylococcus aureus, Staphylococcus epidermidis, Bacillus subtilis, Enterococcus faecalis*) and four Gram-negative (*Escherichia coli, Acinetobacter baumannii, Klebsiella pneumoniae, Citrobacter freundii*). Bacteria collected at the stationary phase of growth (15 h) were washed twice with PBS, resuspended in fresh PBS, and adjusted to OD_600_ = 4 for further use. Dye-loaded nanoparticles solutions were prepared with OD_400_ = 0.2. Pure bacterial samples were used as prepared. To obtain mixed samples, bacterial cultures with OD_600_ = 4 were mixed in corresponding proportions (v/v) before mixing with dye-loaded nanoparticles. Bacterial samples (either pure or mixed) and nanoparticles solutions were mixed 1:1, stirred on a vortex mixer, and incubated in dark for 15 min. All samples were subsequently centrifuged at 15,000 rpm for 1 min, washed once with PBS, and resuspended in the same volume of fresh PBS, keeping the original concentration. Samples of each bacterium or mixture with every dye were then plated on black 96-well plates (150 μL/well, 10 replicates per sample). Emission intensities of the samples at three channels (485, 515, and 575 nm) were recorded at λ_excitation_ = 400 nm using Tecan Infinite 200 spectrofluorometric plate reader. Five independent measurements in each channel were averaged and used to calculate ratiometric responses for further analysis.

### Data Analysis

Fluorescence intensities in three channels were converted into ratiometric signals, and data matrices were prepared that contained 8 unique signals for every data point. The linear discriminant analysis was performed using the XLSTAT software (Addinsoft, NY). Support vector machine analysis was performed using Orange software (Demšar et al., 2013).

## Results and Discussion

### Mixed Samples in the Linear Discriminant Space

The spectrum of a system whose components do not interact with each other constitutes a linear combination of the spectra of the mixture's components. The main hypothesis driving our approach is that this linear relationship will be preserved in the linear discriminant subspace. Indeed, such trends have been observed earlier for various linear progressions, like increasing concentrations of a single analyte (Tao and Auguste, 2016; Zhang et al., 2017) or binary mixtures with varying proportions of the components (Tao and Auguste, 2016; Zheng et al., 2019).

In this study, we used eight bacterial species to test our approach: four Gram-positive (*S. aureus, S. epidermidis, B. subtilis, E. faecalis*) and four Gram-negative (*E. coli, A. baumannii, K. pneumoniae, C. freundii*). A sensor array consisting of four environmentally-sensitive derivatives of 3-hydroxyflavone, described in detail in our previous work (Svechkarev et al., 2018b), was used to generate the fluorescent signals from bacterial samples. The reporter dyes have the same fluorescent core, but various substituents that drive their interactions with different components of the bacterial cell wall. The dyes change their fluorescent spectra in response to universal (polarity) and specific (hydrogen bonding) interactions, thus creating a unique “fingerprint” response pattern for every bacterial species analyzed.

**Figure 1:**
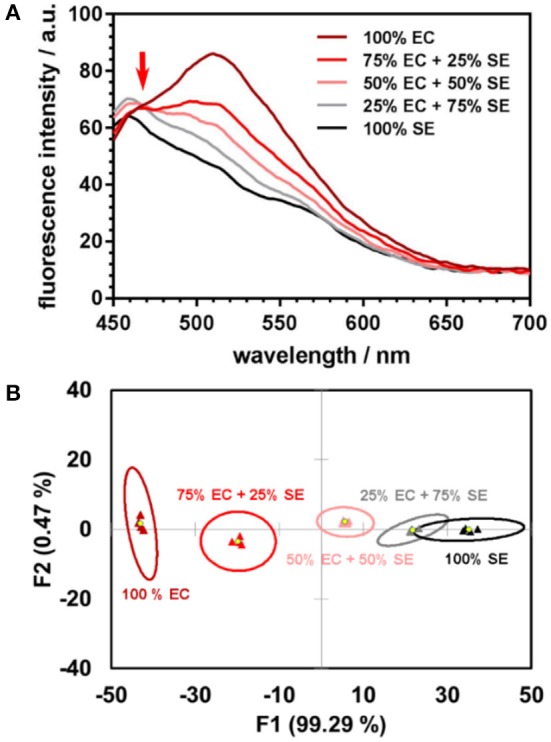
**(A)** Fluorescence spectra of DOAF-loaded nanoparticles upon incubation with *E. coli* (EC), *S. epidermidis* (SE), and their mixtures in different proportions each line is the average of four spectra. The isoemissive point at ~470 nm serves as evidence of the mixed samples spectra being linear combinations of the emission spectra of the pure components. **(B)** Canonical score plot from LDA analysis of response patterns of pure bacteria and their mixtures. Signals from the mixtures are positioned along the line connecting the centroids of the 95% confidence ellipses for the pure bacteria.

In our proof-of-concept experiment, pure samples of *E. coli, S. epidermidis*, and their three different mixtures in various proportions, were investigated. The linear relationship between the spectral responses of the mixtures and those of individual components is evident from the fluorescence spectra (Figure 1A). The linear trend is preserved in the subspace of the first two linear discriminants (Figure 1B). Any departure from linearity may be due to the differences in interaction dynamics of the nanoparticles and/or encapsulated dyes with bacteria, which lead to the dyes being unequally distributed among the mixture's components. Further studies of the nanoparticle-bacteria interactions are underway to perform a detailed analysis of the factors that contribute to deviations from the linear trend.

**Figure 2:**
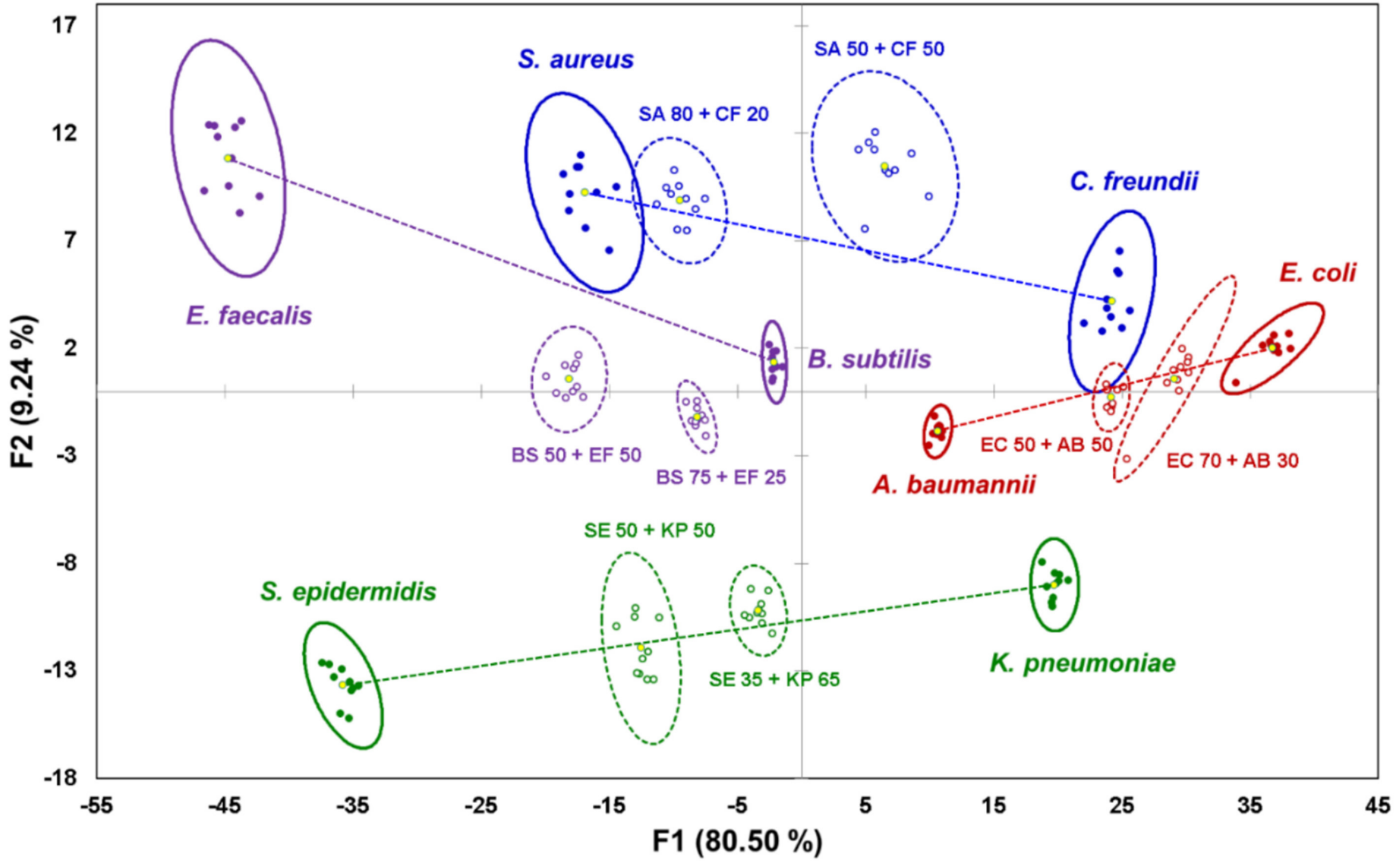
Canonical score plot of the results of LDA for pure bacterial samples and their binary mixtures. Signals from pure bacteria are represented by filled dots and solid ellipses; mixtures are represented by circles and dashed ellipses. Yellow dots are centroids of the 95% confidence ellipses. Lines connecting centroids of the mixture components serve as visual guide.

To investigate the behavior of complex samples upon interaction with the sensor, four pairs of bacterial cells were selected from the species indicated above: one Gram-positive pair, one Gram-negative pair, and two “mixed” pairs with one Gram-positive and one Gram-negative component (Figure 2). For each of the four pairs, two mixtures were prepared: a “standard” 50:50 (v/v) mixture with equal content of both components, and a “random” mixture with an arbitrary composition to test the sensor's quantification abilities. As described previously (Svechkarev et al., 2018b), the sensor array can distinguish the Gram status of the bacterial samples: signals from the Gram-positive bacteria are located in the negative part of the plot along the F1 axis, whereas those from Gram-negative bacteria are found on the positive side. Most of the signals from the mixed samples are located along the lines connecting the ellipse centroids of their respective components—however, some departures from linearity are observed for three of the eight studied mixtures. Locations of the signals on the plot are proportional to the composition of the mixtures: the larger is the content of a given component, the closer the mixture's signal is to the signal of that component.

Classification of unknown samples is a common application of sensor arrays. These systems use a reference dataset, or “training dataset,” that combines the sensor's responses from known analytes, and creates a corresponding canonical score plot similar to one shown in Figure 2. A response from an unknown sample is then analyzed and compared to the responses from the training dataset (Rana et al., 2016). An important consideration is that unknown samples can only be correctly recognized if the signals they generate are already “known” to the sensing system—i.e., they are present among the samples of the training dataset. In this case, the sensor compares and classifies the unknown samples, whereas its prediction capabilities are limited.

### Mixture Composition Quantification

The commonly used classification approach presents a limitation for mixed samples analysis: the system needs to be “trained” to recognize the mixtures by including their response patterns into the training dataset. This significantly increases both the effort needed to create the latter, and its size. Our solution to this problem is to use the linear trends observed for the mixture responses relative to their components. Whereas in traditional classification, the Mahalanobis distances are used to calculate the probability of an unknown sample to belong to a certain class from the training dataset, in our approach we use similar distances—i.e., those between the ellipse centroids—as a measure of the component proportions in binary mixtures.

**Figure 3:**
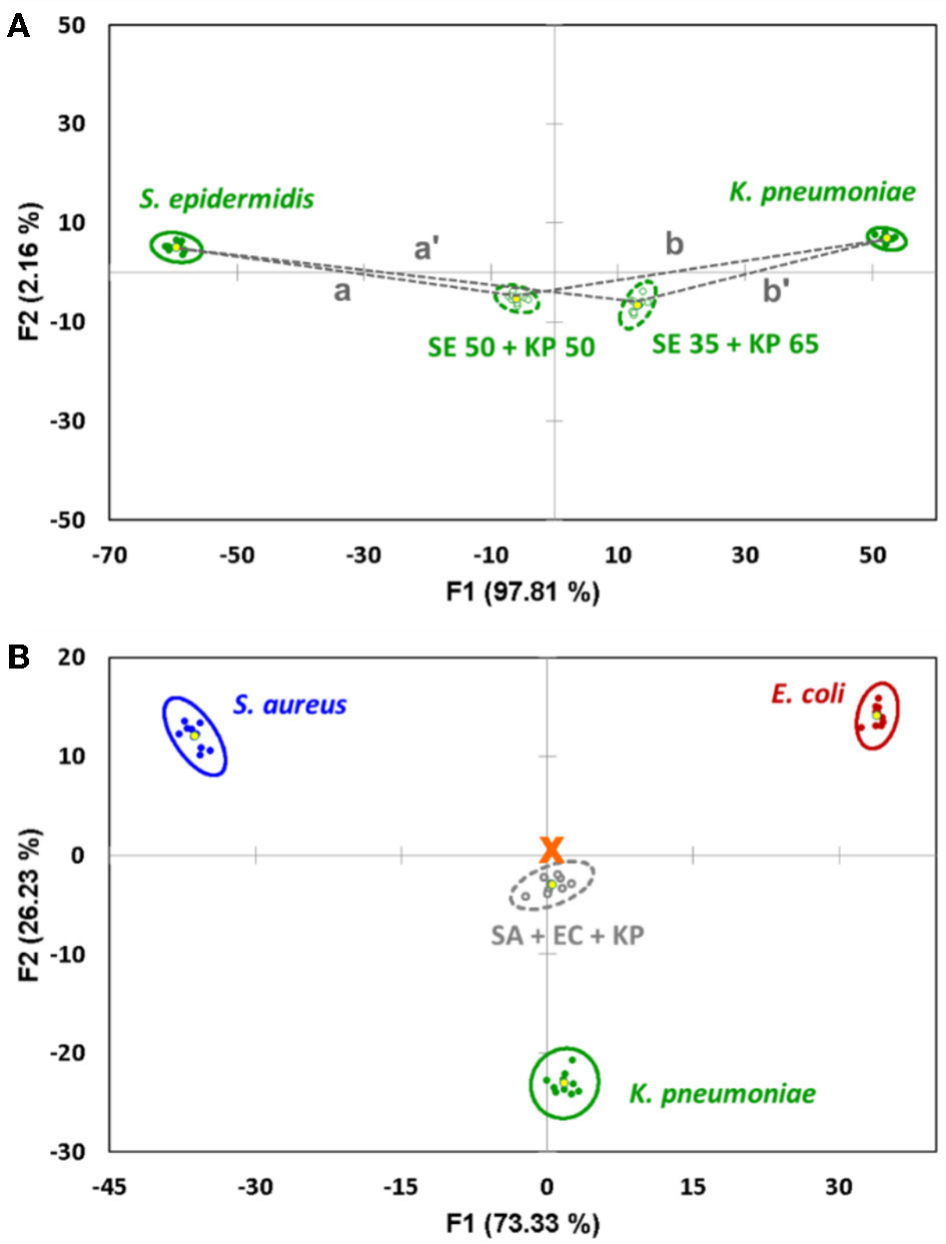
**(A)** Quantification of the binary mixture of bacteria after the components are identified. The content of *S. epidermidis* (SE) is proportional to the distance between the 95% confidence ellipse centroids of the mixture and *K. pneumoniae* (KP). **(B)** Canonical score plot of the sensor's response to an equal mixture of *S. aureus* (SA), *E. coli* (EC) and *K. pneumoniae* (KP) related to the signals of pure components. The signal of the mixture represents an almost perfect linear combination, being located very close to the center of the triangle (marked with an orange cross).

Indeed, we show that the distances between the centroids are proportional to the mixture's composition (Figure 3A), and the content of both components can be estimated using the formulae:

(1) $$\begin{array}{l} X\left(SE\right)=\frac{b}{a+b},\;X\left(KP\right)=\frac{a}{a+b} \end{array}$$

The results of such quantification for all eight studied mixtures are presented in Table 1, columns 3–4. The quantification error in our method mostly varies in the range of 1–8%, with only two exceptions where the observed deviation from the linear trend is also the most significant.

**Table 1:** Component quantification (see Supplementary Table 2 for detailed LDA quantification results).

**Mixture components**	**Proportions prepared, % (v:v)**	**LDA**		**SVM**	
		**Proportions found, %**	**Error**	**Proportions found, %**	**Error**
*B. subtilis +* *E. faecalis*	50:50	64.0:36.0	14.0%	79.6:20.4	29.6%
	75:25	85.5:14.5	10.5%	81.7:18.3	6.7%
*E. coli +* *A. baumannii*	50:50	51.6:48.4	1.6%	70.8:29.2	20.8%
	70:30	70.7:29.3	0.7%	81.7:18.3	11.7%
*S. aureus +* *C. freundii*	50:50	44.5:55.5	5.5%	87.5:12.5	37.5%
	80:20	82.1:17.9	2.1%	91.8:8.2	11.8%
*S. epidermidis +* *K. pneumoniae*	50:50	58.1:41.9	8.1%	27.6:72.4	22.4%
	35:65	41.5:58.5	6.5%	25.9:74.1	9.1%
Average error		6.1 ± 4.4%		18.7 ± 10.1%	

A more advanced method of pattern analysis, support vector machines (SVM), is also routinely used for supervised classification (Askim et al., 2016; Tomberg et al., 2019). In the case of a mixture, its response can be compared to the training set comprising the signals of the components. This gives the probabilities for the mixture signal to be attributed to one of the components. However, the SVM method is not linear by its nature, and thus the linear relationship between the response of the mixture and its components may not be preserved. It is manifested, in particular, in significantly higher quantification errors (Table 1, columns 5–6), where the method shows a notable bias toward the most abundant component.

It is worth noting that the linear trends discussed above for the binary systems are observed for more complex samples as well. Thus, a proof-of-concept measurement with a triple-component mixture of *S. aureus, E. coli*, and *K. pneumoniae* in equal proportions (33.3% v/v each, Figure 3B) showed a near-perfect response, putting the signals from the mixture almost precisely in the center of the triangle formed by the signals from the three individual components. This strongly supports the ability of such sensor arrays to analyze complex multicomponent systems.

### Prediction of Mixture Components

**Figure 4:**
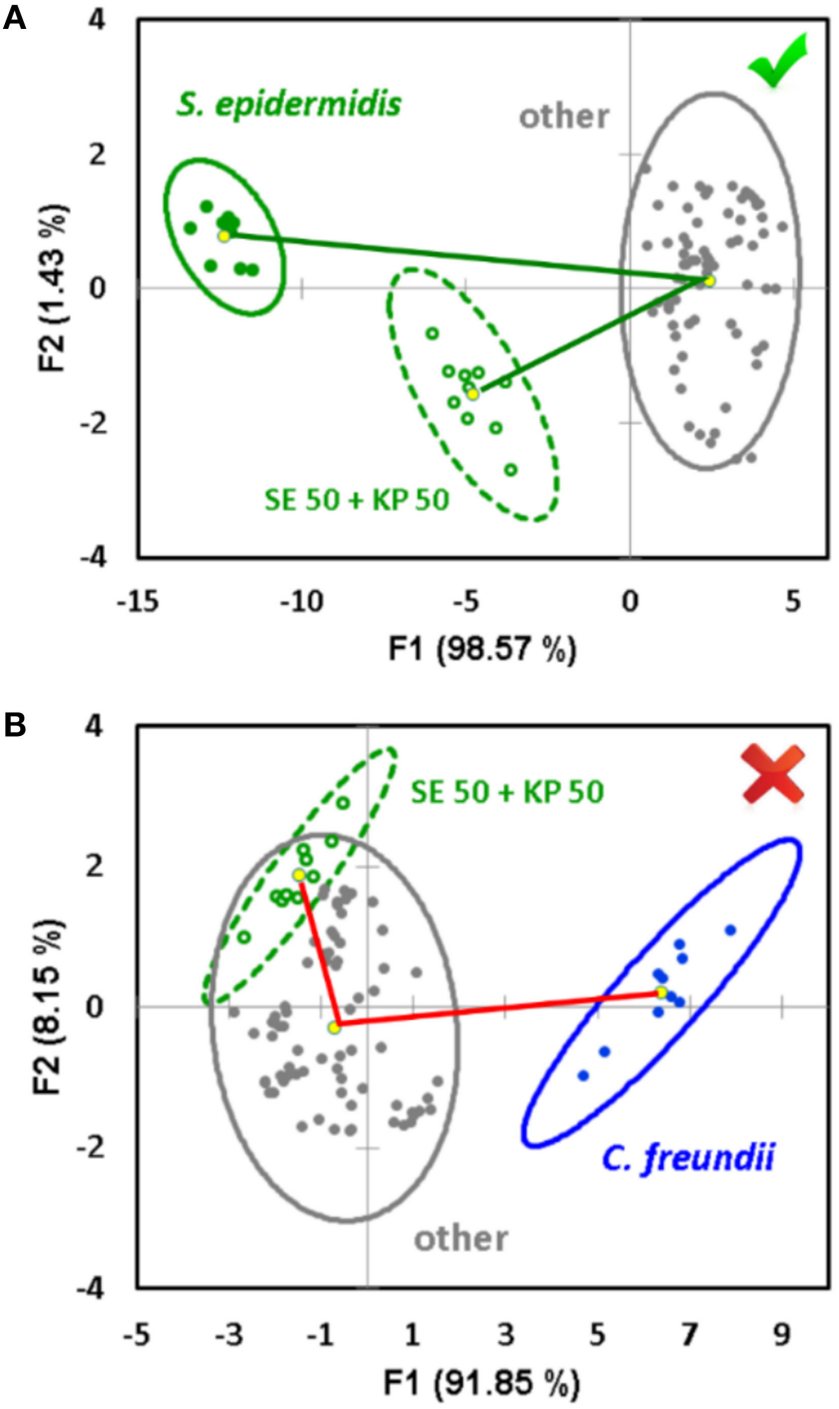
Example of a positive **(A)** and negative **(B)** decision regarding a potential component of an unknown mixture in the “one against the rest” analysis. The decision is made based on the overlap between the 95% confidence ellipses of the mixture and the complex of “other” bacteria, and the angle between the vectors connecting the ellipse centroid of the “other” aggregate group and the signal of the component under question, respectively.

The described approach for the quantification of the components of mixed samples works when the components of an unknown mixture are identified, and they both belong to the training dataset. In addition, our study shows that the sensor array is capable of predicting the components of a mixed sample if they are not known in advance—i.e., without the need of expansion of the training dataset. In order to accomplish this task, a “one against the rest” analysis is used (Schoelkopf and Smola, 2002; Li et al., 2006; Svechkarev et al., 2018b). In this case, the training dataset consists of only two groups of signals: those of a component under question, and those of all other bacterial species combined together (the “other” class). We expect the system to be able to recognize a similarity in the response pattern of the mixture with both the component under question and the “other” bacteria, or only with the “other” class. The first case will mean a positive identification—the component is present in the mixture—and the signal from the mixture will be located somewhere between those of the identified component and the “other” bacteria (Figure 4A). The latter case means that both mixture components belong to the “other” bacteria class, and the component under question is not part of the mixture. It is usually visualized as a significant overlap of the ellipses for the mixture and “other” class signals (Figure 4B). The angle between the lines connecting the ellipse centroid of the “other” class response with the mixture and component under question can be an additional verification of the prediction accuracy: it is <90° in case of a positive attribution, and usually exceeds 90° in case of a negative result (Figure 4).

The “one against the rest” analysis can also be performed using the SVM method instead of LDA. In this case, a probability for the mixture signal to be associated with either the component under question, or other bacteria, will be obtained. The probability data is presented in Supplementary Table 3, and the average results are reported in Table 2 (column 5). A probability of >75% is considered a positive result, <35% is a negative result, and any value in between is considered equivocal. A summary of the prediction accuracy based on correct hits is presented in Table 2, column 4. Overall, the results of both classification methods (LDA and SVM) are very similar, and the prediction accuracy generally exceeds 80%. This compares well with several other methods that demonstrated specificity in the range of 70–85% (Herreros et al., 2015; De Rosa et al., 2018).

**Table 2:** Component prediction accuracy based on “one against the rest” analysis (see Supplementary Figures 2–9 for detailed LDA “one against the rest” classification).

**Mixture components**	**Proportions (v:v)**	**LDA**	**SVM**	
		**Accuracy (hits)^**a**^**	**Accuracy (hits)^**a**^**	**Accuracy (probability)^**b**^**
*B. subtilis +* *E. faecalis*	50:50	6/8 (75%)	7/8 (87.5%)	84.4%
	75:25	7/8 (87.5%)	7/8 (87.5%)	84.8%
*E. coli +* *A. baumannii*	50:50	6/8 (75%)	6.5/8 (81.3%)	81.4%
	70:30	7/8 (87.5%)	7/8 (87.5%)	84.0%
*S. aureus +* *C. freundii*	50:50	5/8 (62.5%)	7/8 (87.5%)	85.2%
	80:20	6/8 (75%)	7/8 (87.5%)	86.3%
*S. epidermidis +* *K. pneumoniae*	50:50	7/8 (87.5%)	5.5/8 (68.8%)	70.6%
	35:65	7/8 (87.5%)	6.5/8 (81.3%)	78.0%
Average		6.4/8 (79.7 ± 8.7%)	6.7/8 (83.6 ± 6.2%)	81.8 ± 4.9%

a A correct hit (positive for a component present in the mixture, negative for a component absent in the mixture) is given 1 point, an ambiguous result is given 0.5 point, an incorrect hit (false positive or negative) is given 0 points.

b *The average of the probabilities calculated for a mixture's response to be associated with every single component against the aggregate of the rest of components*.

The new approach to the processing of sensor array responses described in this report significantly expands the abilities of such sensors in both detection, prediction, and even quantification of complex samples, without the need for acquisition of new reference data and expansion of the training datasets. Further studies of the interaction dynamics of the reporter dyes and bacterial cell walls will allow for improved accuracy of this sensor array approach.

## Data Availability Statement

The datasets generated for this study are included in the article/Supplementary Material, and can be obtained from the authors by request.

## Author Contributions

DS conceptualized the project and prepared the original draft. DS and MS developed the methodology and performed the experiments. DS and LH prepared the sensor arrays and analyzed the data. KB and AM supervised the project. All authors participated in the review and editing of the manuscript.

### Conflict of Interest

The authors declare that the research was conducted in the absence of any commercial or financial relationships that could be construed as a potential conflict of interest.
