# Supplementary material for: Fluorescent Sensor Arrays Can Predict and Quantify the Composition of Multicomponent Bacterial Samples
Source: Front Chem. 2020 Jan 15;7:916. doi: 10.3389/fchem.2019.00916 (PMC6974461; doi:10.3389/fchem.2019.00916)
Supplement: Supplementary file 1 [file Data_Sheet_1.PDF]

Supplementary Material

1 Synthesis of the dyes

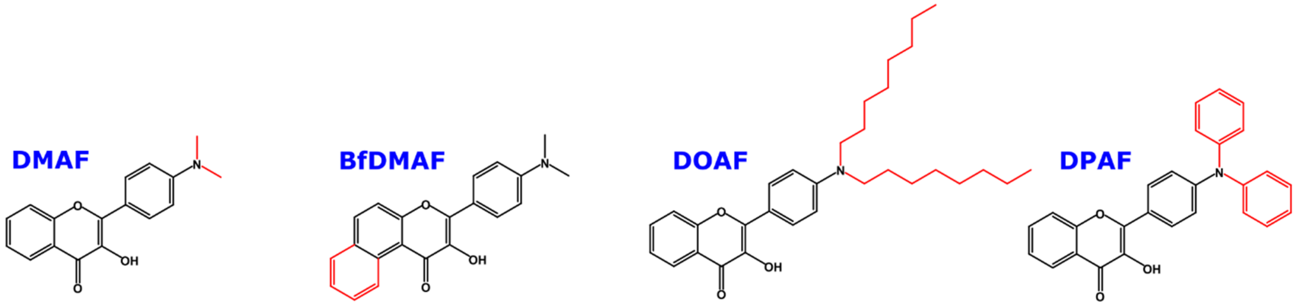

All four derivatives of 3-hydroxychromone were synthesized following a two-stage Algar-Flynn-Oyamada procedure modified for better performance with electron donor-substituted aldehydes [S1]. To 1 g of a N,N-disubstituted aminobenzaldehyde, 1 equiv. of 2-hydroxyacetophenone (or 2-hydroxynaphthophenone to obtain BfDMAF) was added in a 50 mL round-bottom flask, and the compounds were dissolved in 10 mL of DMF. Subsequently, 3 equiv. of sodium methylate were added, and the mixture was stirred at room temperature for 3 hours. After that, the mixture was transferred into a bigger flask and diluted with 50 mL of ethanol. An excess of 30 equiv. of sodium methoxide was then added, followed by an excess of 20 equiv. of hydrogen peroxide (30% v/v), that was added dropwise while stirring. Formation of the gel of the intermediate epoxide product was observed, which then dissolved upon further addition of H_2O_2. The mixture was subsequently refluxed for 2-3 minutes and left to cool down. After that, the reaction mixture was diluted with 50 mL of ultrapure water, and neutralized to pH 6-7. The product was filtered (extracted with chloroform in case of DOAF) and purified as indicated below. structures of the obtained compounds were confirmed by 1H NMR spectroscopy on a Bruker Advance 500 MHz NMR spectrometer at 25°C. Samples were analyzed in DMSO-d_6 (99.9% D, Cambridge Isotope Laboratories). Data was processed in Mnova NMR (Escondido, CA).

DMAF: the crude product was recrystallized from methanol. Yield 0.71 g (35.5 %). ^{1}H NMR (500 MHz, DMSO-d_6): 3.01 (s, 6H), 6.84 (d, 2H), 7.42 (t, 1H), 7.73 (m, 2H), 8.07 (d, 1H), 8.12 (d, 2H), 9.16 (s, 1H).

BfDMAF: the crude product was recrystallized from methanol. Yield 0.92 g (41.5 %). ^{1}H NMR (500 MHz, DMSO-d_6): 3.02 (s, 6H), 6.87 (d, 2H), 7.66 (t, 1H), 7.77-7.85 (m, 2H), 8.08 (d, 1H), 8.16 (d, 2H), 8.27 (d, 1H), 9.23 (s, 1H), 10.01 (d, 1H).

DOAF: the crude product was purified by column chromatography (silica gel, chloroform). Yield 0.31 g (22.2 %). ^{1}H NMR (500 MHz, DMSO-d_6): 0.84 (t, 6H), 1.24-1.27 (m, 20H), 1.47 (m, 4H), 3.20 (t, 4H), 6.50 (t, 1H), 6.59 (d, 2H), 6.71 (d, 1H), 7.07 (t, 2H), 7.69 (d, 1H), 8.07 (d, 1H), 9.60 (s, 1H).

DPAF: the crude product was recrystallized from methanol. Yield 0.51 g (34.7 %). ^{1}H NMR (500 MHz, DMSO-d_6): 7.04 (d, 2H), 7.14 (m, 6H), 7.37 (m, 4H), 7.45 (t, 1H), 7.70 (d, 1H), 7.78 (t, 1H), 8.09 (t, 3H), 9.31 (s, broad, 1H).

2 Dye loading quantification

Dye loading quantification was performed using UV/vis absorption spectroscopy. Calibration graphs for all four dyes in DMSO were obtained (Supplementary Figure 1). After that, absorptions of dye-loaded NP solutions with known concentrations in DMSO were recorded, and the content of the dye in the dye-loaded NP samples were calculated Supplementary Table 1). DMSO was used to ensure the disassembly of NPs in order to avoid dye aggregation affecting the calibration and quantification data.

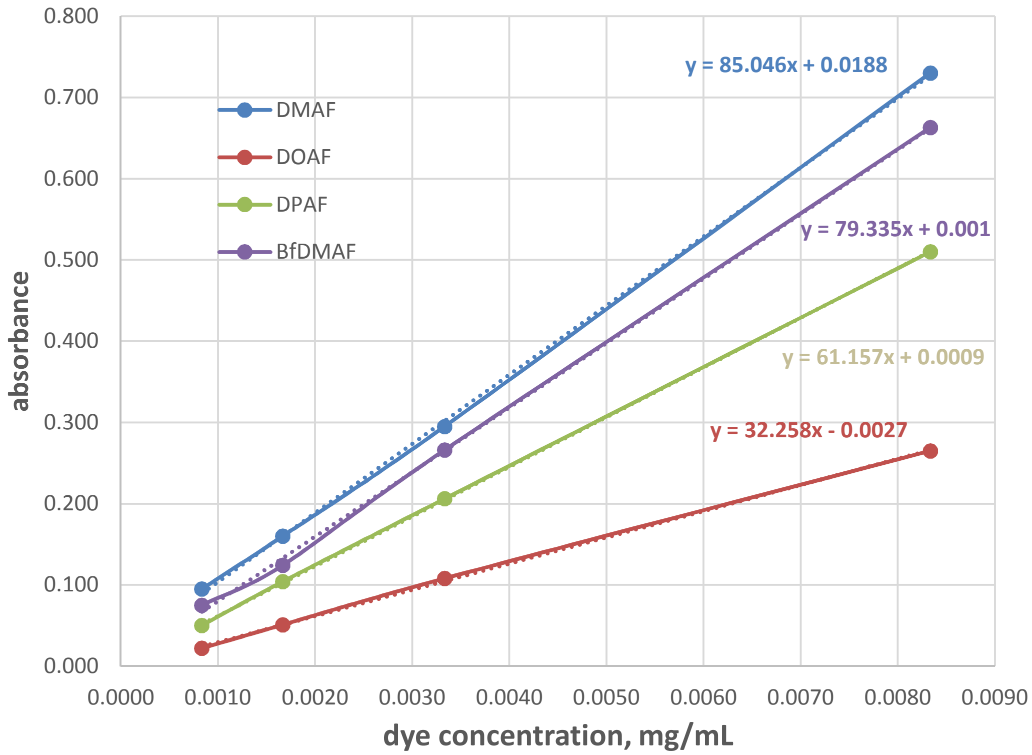

Supplementary Figure 1. Calibration data for the dye content quantification in the dye-loaded HA NPs (λ_{abs} = 400 nm).

Supplementary Table 1. Dye content in the dye-loaded NPs.

Dye	Content, % (w/w)
DMAF	0.3
BfDMAF	2.6
DOAF	1.3
DPAF	0.7

3 Canonical score plots of the LDA results for the "one against the rest" analysis to predict the components of the unknown binary mixtures

Each canonical score plot represents an LDA treatment of the mixture's response in comparison with the component under question (bacterial species that will be either confirmed or rejected as the mixture's component), and the rest of bacterial species from the training dataset. LDA processing was performed using XLSTAT software [S2].

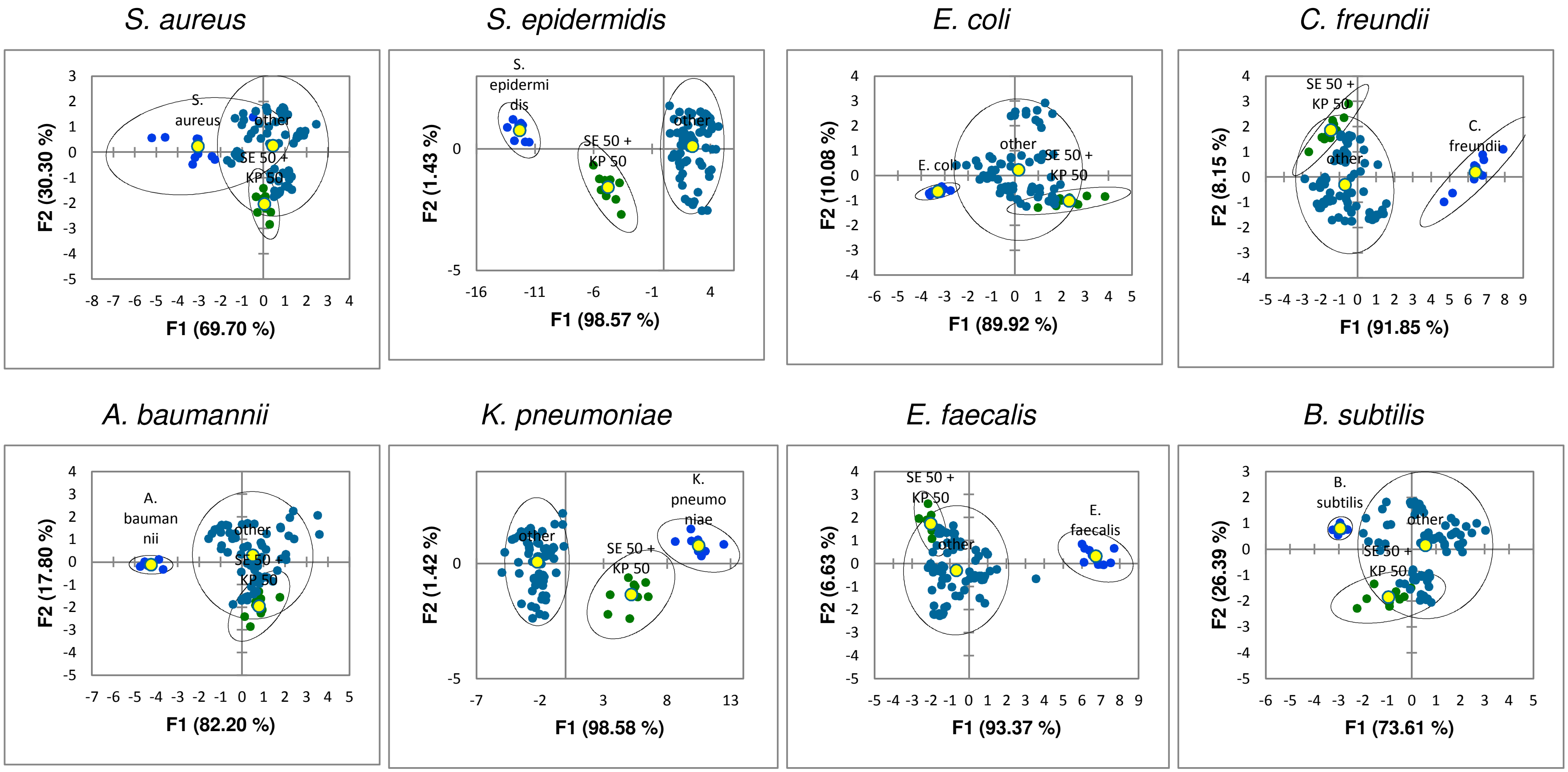

Supplementary Figure 2. Prediction of the components of the mixture of S. epidermidis and K. pneumoniae (50:50 v/v) using "one against the rest" approach in the linear discriminant analysis.

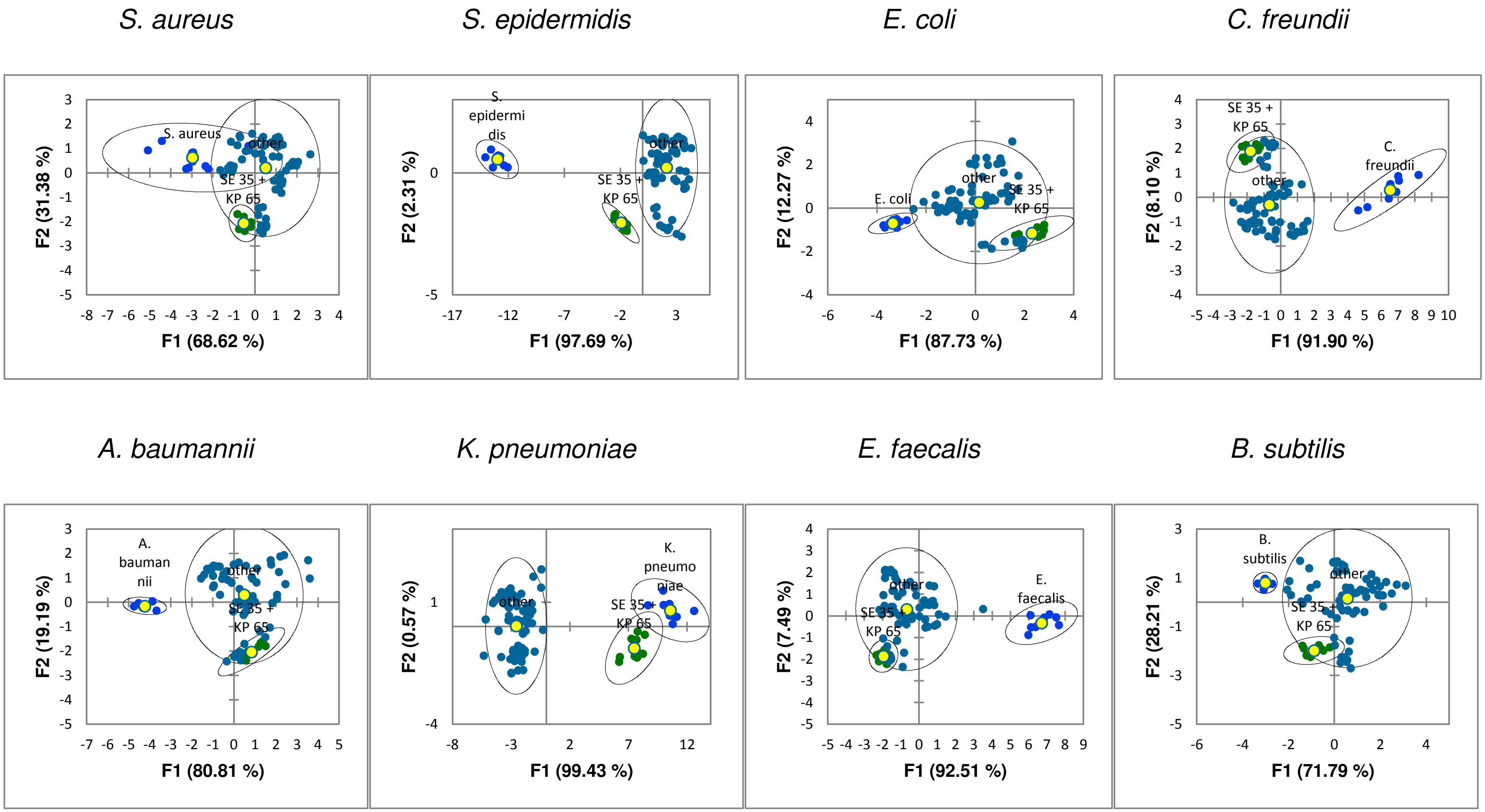

Supplementary Figure 3. Prediction of the components of the mixture of S. epidermidis and K. pneumoniae (35:65 v/v) using “one against the rest” approach in the linear discriminant analysis.

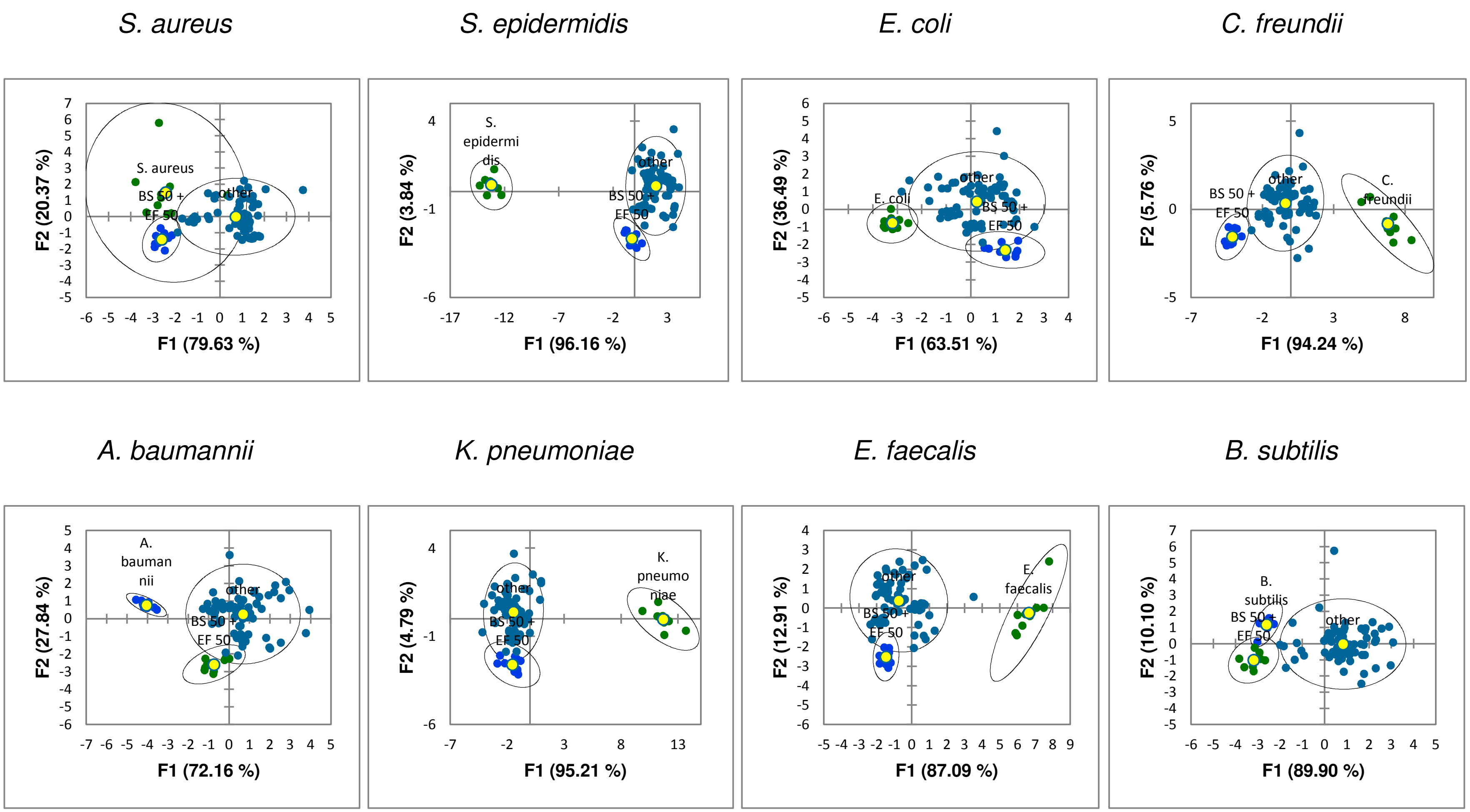

Supplementary Figure 4. Prediction of the components of the mixture of B. subtilis and E. faecalis (50:50 v/v) using “one against the rest” approach in the linear discriminant analysis.

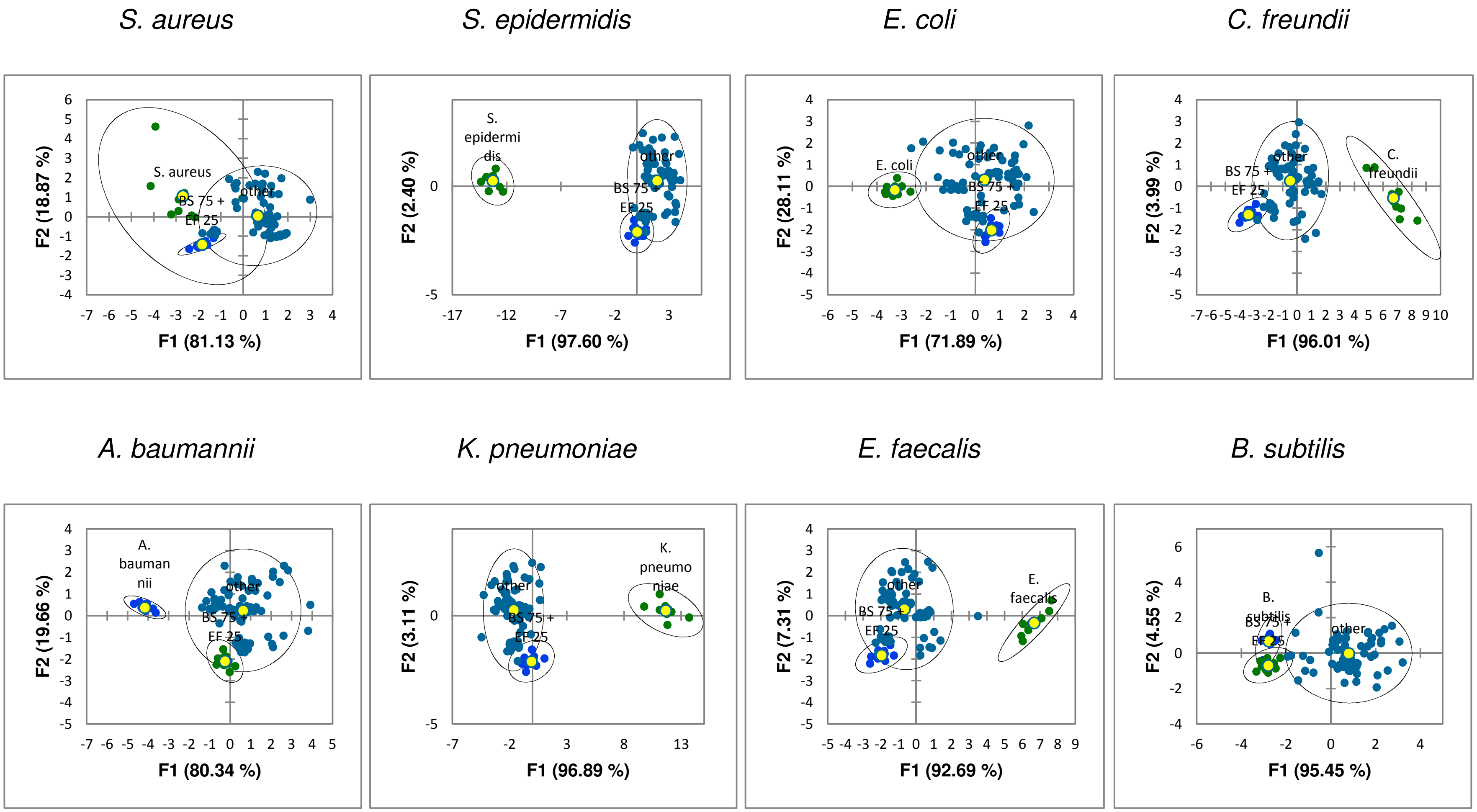

Supplementary Figure 5. Prediction of the components of the mixture of B. subtilis and E. faecalis (75:25 v/v) using “one against the rest” approach in the linear discriminant analysis.

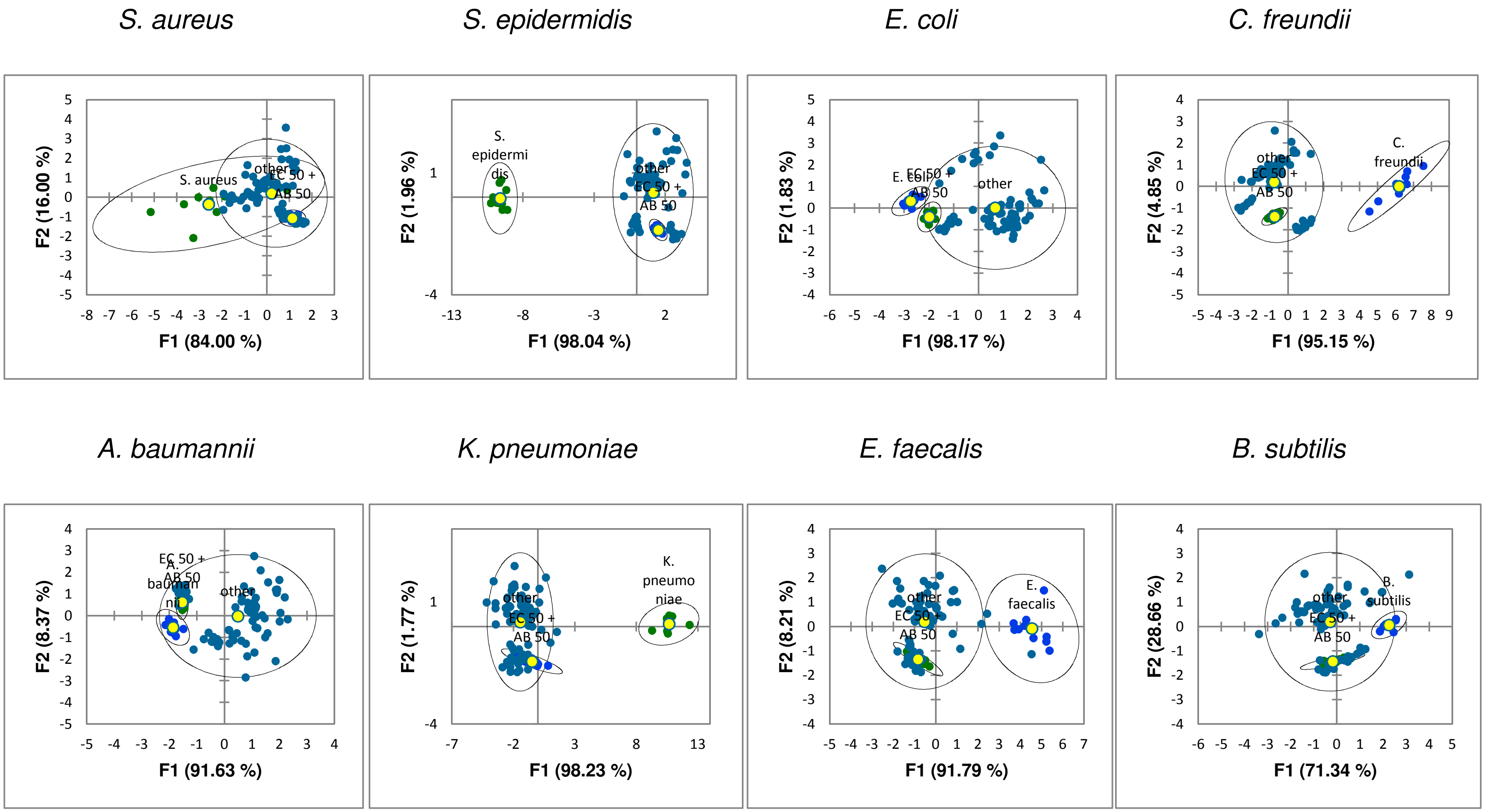

Supplementary Figure 6. Prediction of the components of the mixture of E. coli and A. baumannii (50:50 v/v) using "one against the rest" approach in the linear discriminant analysis.

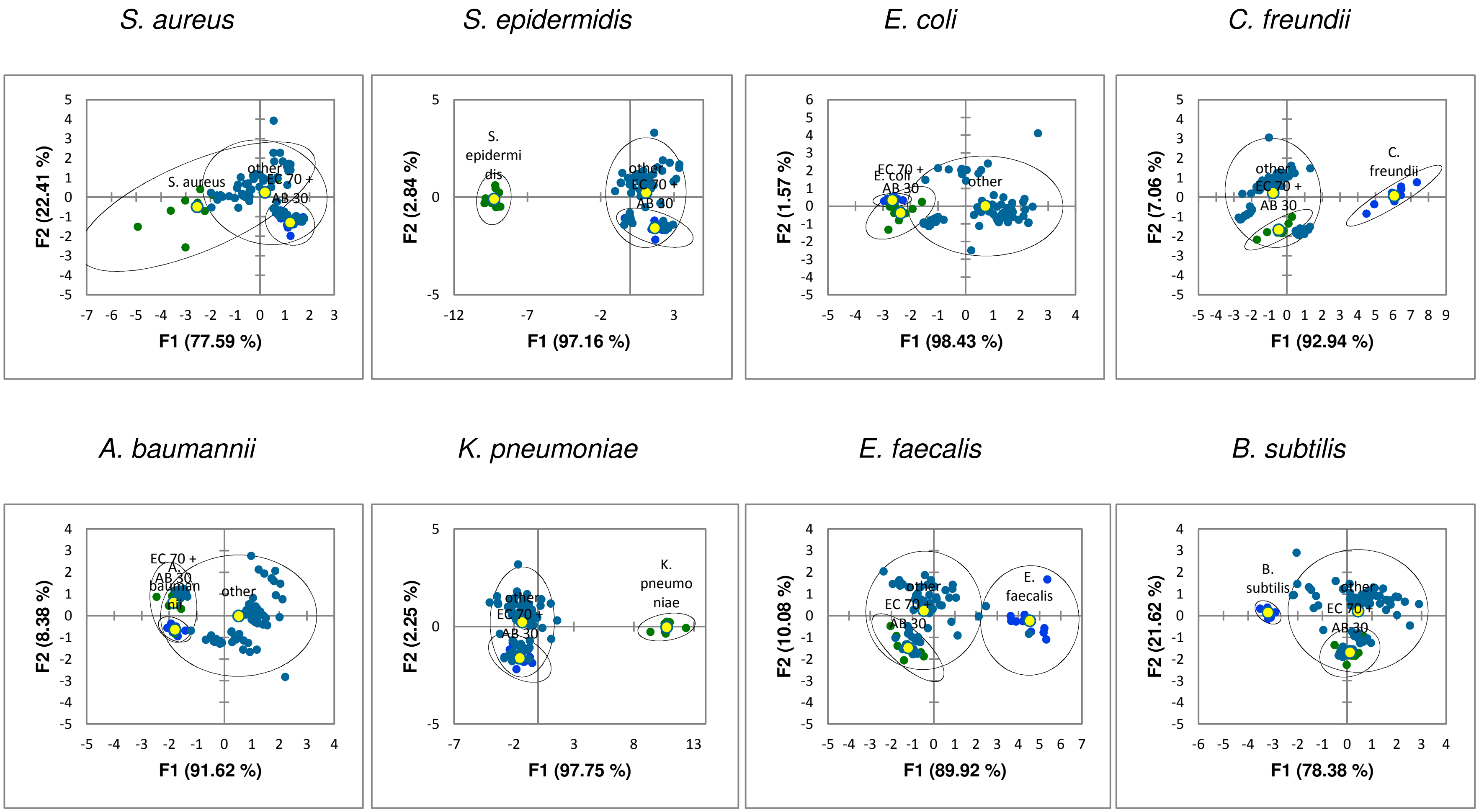

Supplementary Figure 7. Prediction of the components of the mixture of E. coli and A. baumannii (70:30 v/v) using "one against the rest" approach in the linear discriminant analysis.

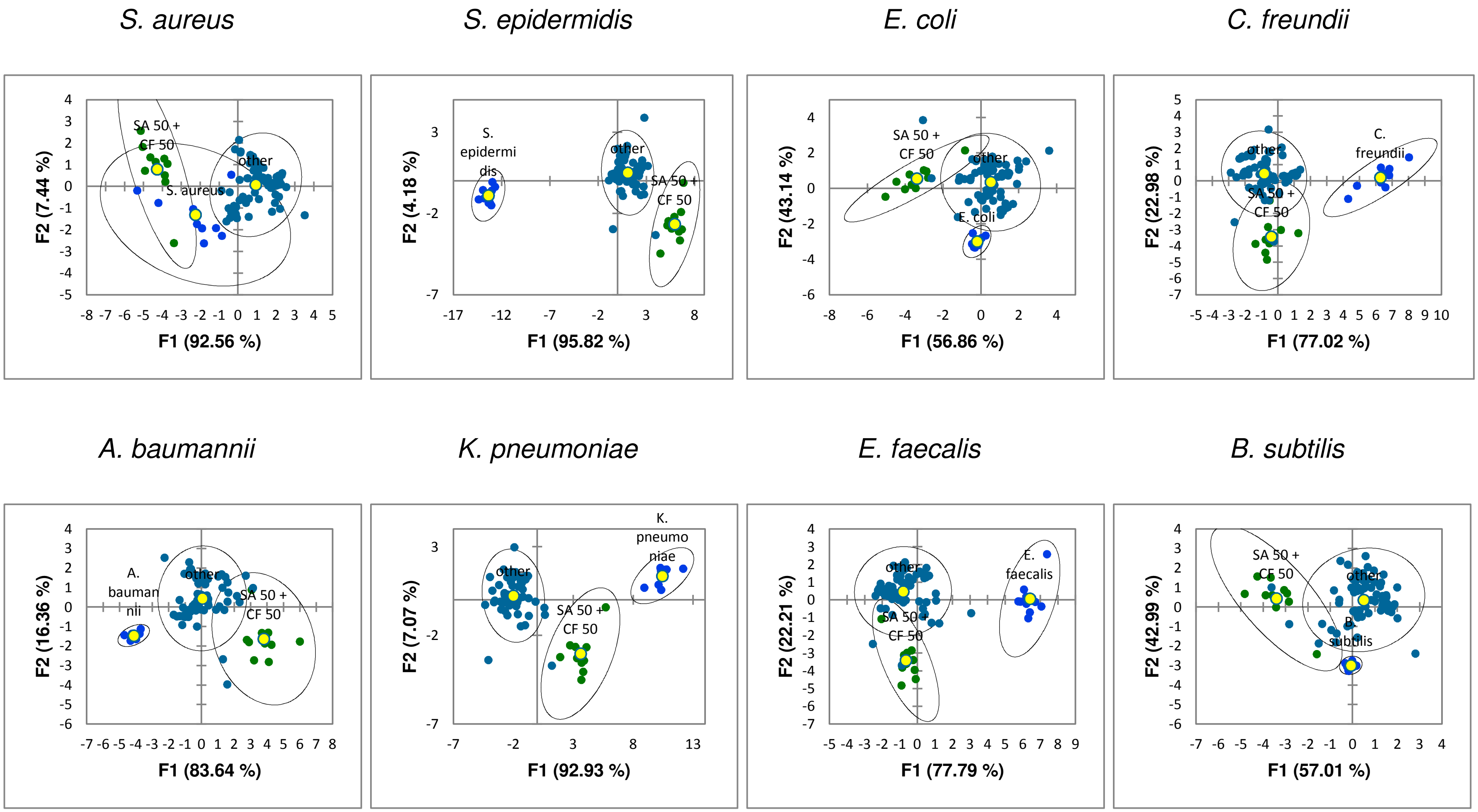

Supplementary Figure 8. Prediction of the components of the mixture of S. aureus and C. freundii (50:50 v/v) using "one against the rest" approach in the linear discriminant analysis.

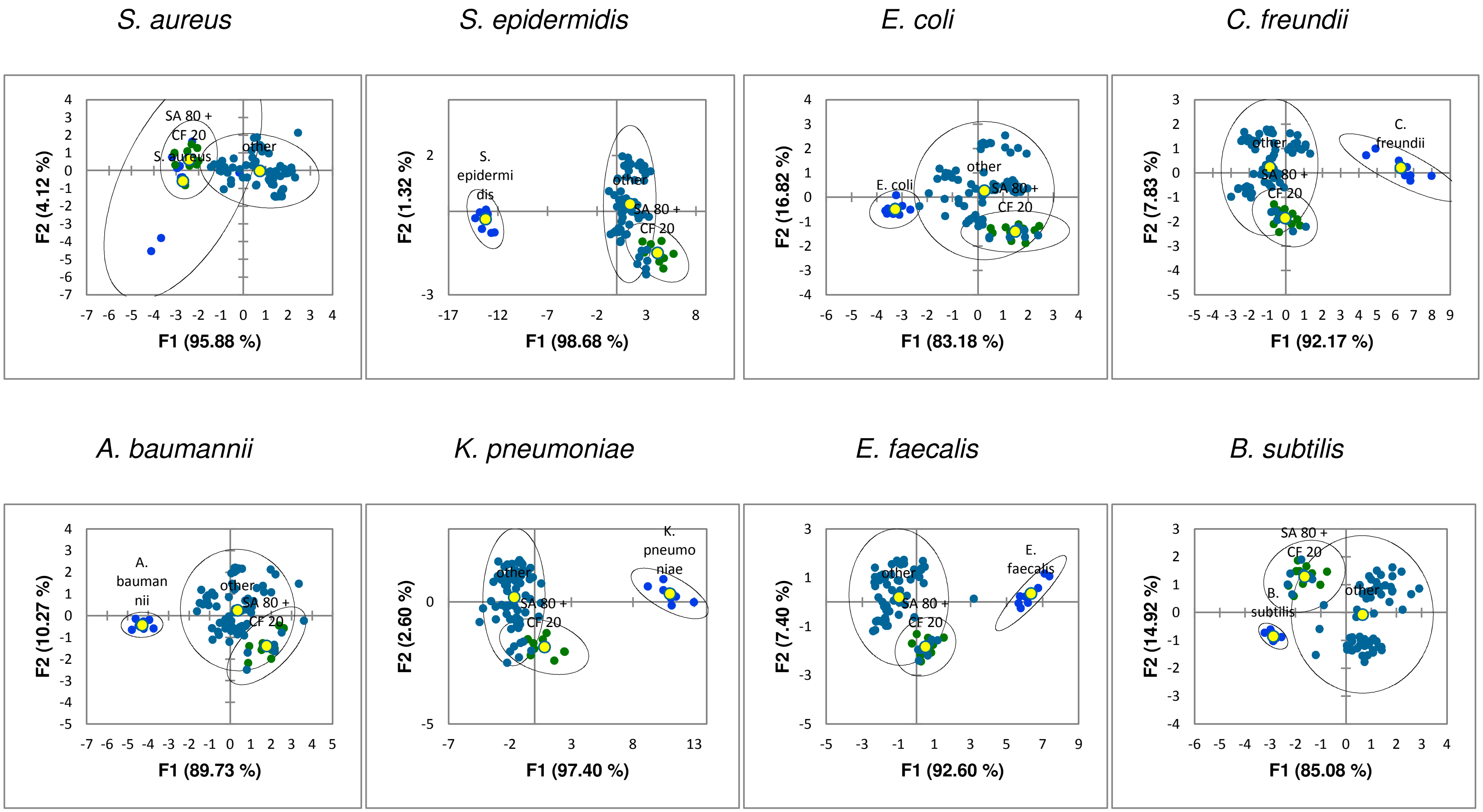

Supplementary Figure 9. Prediction of the components of the mixture of S. aureus and C. freundii (80:20 v/v) using “one against the rest” approach in the linear discriminant analysis.

4 Quantification of the mixture composition using centroid coordinates from the results of LDA processing

Supplementary Table 2. Centroid coordinates and results of mixture composition quantification.

mixtures	centroid comp 1		centroid comp 2		centroid mixture		found		error
	x	y	x	y	x	y	1	2	
BS+EF 50-50	-2.126	1.363	-44.721	10.843	-18.123	0.562	64.0%	36.0%	14.0%
BS+EF 75-25	-2.126	1.363	-44.721	10.843	-8.146	-1.184	85.5%	14.5%	10.5%
EC+AB 50-50	36.749	2.005	10.631	-1.859	24.171	-0.276	51.6%	48.4%	1.6%
EC+AB 70-30	36.749	2.005	10.631	-1.859	29.129	0.594	70.7%	29.3%	0.7%
SA+CF 50-50	-16.907	9.243	24.234	4.180	6.512	10.462	44.5%	55.5%	5.5%
SA+CF 80-20	-16.907	9.243	24.234	4.180	-9.508	8.873	82.1%	17.9%	2.1%
SE+KP 50-50	-35.766	-13.672	19.746	-8.998	-12.512	-11.941	58.1%	41.9%	8.1%
SE+KP 35-65	-35.766	-13.672	19.746	-8.998	-3.364	-10.195	41.5%	58.5%	6.5%

5 Probability data for the "one against the rest" analysis using the support vector machines method for mixtures component prediction

Like in the case of LDA processing presented above, for every mixture each of the 8 possible components was tested as a potential component against the rest of bacteria in the training dataset. Unlike with LDA, instead of the graphical output the SVM method produced the probabilities of each of 10 replicates of every mixture to be associated with either a component under question or the other bacteria. Probabilities of a correct attribution for the replicates were averaged and are presented in Table S1. For the true mixture components (framed for each mixture), a probability of positive attribution is shown. For the species that are not part of the mixture, the probability of a negative attribution (i.e., association with the "other" class) is shown. Green fill of the cell represents a correct analysis result, red fill represents a false positive or a false negative, yellow fill represents an equivocal result. SVM analysis was performed using Orange software [S3].

Supplementary Table 3. Probability data for the mixture components prediction using SVM.

mixtures	AB	BS	CF	EC	EF	KP	SA	SE	accuracy	hits
BS+EF 50-50	98.4%	86.6%	98.7%	99.0%	1.3%	99.3%	98.4%	93.7%	84.4%	87.5%
BS+EF 75-25	98.4%	86.9%	98.5%	99.0%	1.6%	97.2%	99.0%	98.0%	84.8%	87.5%
EC+AB 50-50	19.1%	96.5%	99.0%	42.8%	98.4%	98.5%	98.0%	99.0%	81.4%	81.3%
EC+AB 70-30	13.1%	98.7%	99.0%	68.3%	98.0%	98.7%	97.2%	99.0%	84.0%	87.5%
SA+CF 50-50	99.1%	95.4%	2.8%	97.4%	98.9%	97.8%	90.7%	99.8%	85.2%	87.5%
SA+CF 80-20	99.0%	96.4%	1.2%	98.3%	99.0%	98.9%	98.7%	99.0%	86.3%	87.5%
SE+KP 50-50	99.8%	50.5%	98.0%	99.0%	98.2%	11.9%	99.0%	8.0%	70.6%	68.8%
SE+KP 35-65	99.1%	78.1%	98.0%	99.0%	98.0%	49.7%	99.0%	3.0%	78.0%	81.3%
								**TOTAL**	**81.8%**	**83.6%**

References

S1. *Klymchenko A. S., Pivovarenko V. G., Demchenko A. P.* Elimination of the Hydrogen Bonding Effect on the Solvatochromism of 3-Hydroxyflavones. J. Phys. Chem. A **107** (2003) 4211– 4216.

S2. Addinsoft (2019). XLSTAT statistical and data analysis solution. Long Island, NY, USA. https://www.xlstat.com.

S3. *Demsar J., Curk T., Erjavec A., Gorup C., Hocevar T., Milutinovic M., Mozina M., Polajnar M., Toplak M., Staric A., Stajdohar M., Umek L., Zagar L., Zbontar J., Zitnik M., Zupan B.* Orange: Data Mining Toolbox in Python. J. Machine Learn. Res. **14** (2013) 2349–2353.
